# Inflammatory properties of inhibitor of DNA binding 1 secreted by synovial fibroblasts in rheumatoid arthritis

**DOI:** 10.1186/s13075-016-0984-3

**Published:** 2016-04-12

**Authors:** Gautam Edhayan, Ray A. Ohara, W. Alex Stinson, M. Asif Amin, Takeo Isozaki, Christine M. Ha, G. Kenneth Haines, Rachel Morgan, Phillip L. Campbell, Ali S. Arbab, Sean C. Friday, David A. Fox, Jeffrey H. Ruth

**Affiliations:** Division of Rheumatology, Department of Internal Medicine and Clinical Autoimmunity Center of Excellence, University of Michigan Medical School, 109 Zina Pitcher Drive, 4023 BSRB, Ann Arbor, MI 48109-2200 USA; Mount Sinai Health System, New York, NY 10019 USA; Henry Ford Hospital, Detroit, MI 48202 USA

**Keywords:** Inhibitor of DNA binding-1 protein (Id1), Inflammation, Rheumatoid arthritis, Fibroblasts, Angiogenesis

## Abstract

**Background:**

Inhibitor of DNA binding 1 (Id1) is a nuclear protein containing a basic helix-loop-helix (bHLH) domain that regulates cell growth by selective binding and prevention of gene transcription. Sources of Id1 production in rheumatoid arthritis synovial tissue (RA ST) and its range of functional effects in RA remain to be clarified.

**Methods:**

We analyzed Id1 produced from synovial fibroblasts and endothelial cells (ECs) with histology and real-time polymerase chain reaction (RT-PCR). Fibroblast supernatants subjected to differential centrifugation to isolate and purify exosomes were measured for Id1 by enzyme-linked immunosorbent assay (ELISA). Western blotting of Id1-stimulated ECs was performed to determine the kinetics of intracellular protein phosphorylation. EC intracellular signaling pathways induced by Id1 were subsequently targeted with silencing RNA (siRNA) for angiogenesis inhibition.

**Results:**

By PCR and histologic analysis, we found that the primary source of Id1 in STs is from activated fibroblasts that correlate with inflammatory scores in human RA ST and in joints from K/BxN serum-induced mice. Normal (NL) and RA synovial fibroblasts increase Id1 production with stimulation by transforming growth factor beta (TGF-β). Most of the Id1 released by RA synovial fibroblasts is contained within exosomes. Endothelial progenitor cells (EPCs) and human dermal microvascular ECs (HMVECs) activate the Jnk signaling pathway in response to Id1, and Jnk siRNA reverses Id1-induced HMVEC vessel formation in Matrigel plugs in vivo.

**Conclusions:**

Id1 is a pleotropic molecule affecting angiogenesis, vasculogenesis, and fibrosis. Our data shows that Id1 is not only an important nuclear protein, but also can be released from fibroblasts via exosomes. The ability of extracellular Id1 to activate signaling pathways expands the role of Id1 in the orchestration of tissue inflammation.

## Background

Rheumatoid arthritis (RA) is a systemic autoimmune disease characterized by inflammation and joint destruction. Angiogenesis is important in a variety of vasculoproliferative states such as wound repair and RA synovitis. Many inflammatory mediators found in RA synovial tissues (STs) and synovial fluids (SFs) display angiogenic properties. Inhibitor of DNA binding 1 (Id1) is a member of the helix-loop-helix (HLH) family of transcription factors and a marker of cellular self-renewal. Inhibition of Id1 in the bone marrow (BM) results in significant decreases in endothelial progenitor cell (EPC)-linked tumor-associated vasculogenesis [1]. Gao et al. showed that it was possible to identify, track, and target BM-derived endothelial EPCs in vivo using a mouse model of pulmonary metastasis by measurement of Id1 expression [1, 2].

Id1 lacks DNA binding activity, but instead forms heterodimers with members of the basic helix-loop-helix (bHLH) family of transcription factors, allowing Id1 to inhibit DNA binding and transcriptional activation of proteins with which it interacts. bHLH proteins such as BMAL1-Clock (circadian locomotor output cycles kaput) [3], which is a core transcription complex in the molecular circadian clock, bind Id1. Id1 also interacts with various genes, such as *c-Myc* [4] and hypoxia-inducible factor-1 (HIF-1) [5], that have been linked to cancer due to their effects on cell growth and metabolism. Id1 binds tightly to ubiquitously expressed E proteins, that heterodimerize with tissue-restricted bHLH proteins to form active transcription complexes. By sequestering E proteins, Id1 inhibits tissue-restricted gene expression in multiple cell lineages using the same biochemical mechanism [6, 7].

The human *Id1* gene has been cloned and characterized by Hara et al. [8], who also cloned a related gene, *Id2*. A splice variant (Id1-prime) that does not show the growth-regulated expression normally seen with Id1 has also been described [9]. The Id1-related proteins Id2 and Id3 [10] are also negative regulators of pluripotent stem cell maturation [11]. As a protein affecting the activity of many transcription factors, Id1 can affect multiple cellular properties. Targeting of Id1 transcription blocks EPC mobilization, causes angiogenesis inhibition, impairs the spread of metastasis, and increases the survival of tumor-bearing mice [1, 2]. Interestingly, human Id1 mRNA is barely detectable in quiescent early-passage fibroblasts, but its expression can be induced by serum. Moreover, Id1 antisense RNA prevents early-passage fibroblasts from entering the S phase of the cell cycle [8].

Histologic analysis of ST revealed that Id1 is highly expressed in the vasculature of RA ST [12], and also in synoviocytes (SNCs). Sakurai et al. also showed substantial expression of Id1 and Id3 in RA compared to osteoarthritis (OA) synovium at the protein and transcriptional levels [13]. Our group was the first to report that RA synovial fluid (SF) contains abundant amounts of Id1, and we now show that the primary source is not from EPCs or endothelial cells (ECs), but from RA ST fibroblasts. We also show that Id1 is packaged within extracellular vesicles (EVs) and released from fibroblasts via exosomes. Lastly, we provide evidence that Id1 activates EC signaling pathways, inducing angiogenic responses [12], which can be targeted to reduce blood vessel growth.

## Methods

### Ethical use of animals

Procedures involving animals in this study were approved by The University Committee for the Use and Care of Animals (UCUCA) at the University of Michigan. Mice were housed in sterile rodent micro-isolator caging with filtered cage tops in a specific pathogen-free environment. Severe combined immunodeficient (SCID) mice were obtained from the National Cancer Institute (NCI). C57/BL6 wildtype (Wt) mice were bred in house. All efforts were made to reduce stress or discomfort in the animals used in these studies.

### Patient samples

STs were obtained from RA patients undergoing total joint replacement who met the American College of Rheumatology criteria for RA. Prior to surgery unrelated to the proposed research, patients were asked whether they were willing to contribute ST to the study. ST specimens were stored at -80 °C. All human specimens were consented for use in this study by the Institutional Review Boards of the University of Michigan Medical School (IRBMED).

### K/BxN serum-induced arthritis model

To generate arthritic K/BxN mice, K/B-positive mice were crossed with NOD/LTj mice as previously described [14]. Naïve C57BL/6 mice at the age of 5–7 weeks were injected with 150 μL of K/BxN serum intraperitoneally, and this was considered day 0 of arthritis. Another injection of 150 μL of K/BxN serum followed on day 2. Robust arthritis with severe swelling of the joints typically developed on day 5. Articular index (AI) scores and joint circumferences were determined starting on day 0 and scored at least every other day up to day 23 after induction of arthritis, as described previously for rat adjuvant-induced arthritis [15]. Clinical scoring for arthritis was performed using a 0–4 AI scale, where 0 = no swelling or erythema, 1 = slight swelling and/or erythema, 2 = low-to-moderate edema, 3 = pronounced edema with limited use of the joint, and 4 = excessive edema with joint rigidity. All measurements were taken by observers blinded to the experimental conditions. Mouse ankles were harvested for histology.

### Immunohistochemisty (IHC)

RA, OA, and normal (NL) (no arthritis) ST cryosections as well as ankle sections of Wt mice induced with K/BxN serum were fixed in cold acetone for 30 min at 4 °C. The tissue sections were blocked with 5 % donkey serum and 20 % fetal bovine serum (FBS) in phosphate-buffered saline (PBS) at 37 °C for 1 h. The sections were then incubated with either mouse anti-human Id1 antibody (Abcam, Cambridge, MA, USA, 10 μg/mL), rabbit anti-mouse Id1 antibody (CalBioreagents, San Mateo, CA, USA, 10 μg/mL), or purified nonspecific mouse and rabbit immunoglobulin G (IgG) (Thermo Fisher Scientific, Waltham, MA, USA) for 1 h at 37 °C in blocking buffer. After washing, tissues were incubated with a biotinylated anti-mouse or anti-rabbit secondary antibody (Vector Laboratories, Burlingame, CA, USA, 10 μg/mL) for 1 h at 37 °C in blocking buffer. Vectastain ABC kit (Vector Laboratories) was used to detect the antibodies on the tissues, following manufacturer’s protocols. Sections were mounted with Cytoseal 60 (Thermo Fisher Scientific), visualized under an Olympus microscope (Olympus, Tokyo, Japan) and scored by a pathologist.

#### Immunofluorescence histology

RA, OA, and NL ST sections were fixed in cold acetone for 30 min. The STs were blocked with 5 % donkey serum and 20 % FBS in PBS at 37 °C for 1 h, and then incubated with rabbit anti-human Id1 antibody (Abcam, 10 μg/ml) or purified nonspecific rabbit IgG for 1 h at 37 °C in blocking buffer. The synovial tissue samples were washed with PBS, and a 1:200 dilution in blocking buffer of fluorescent-conjugated donkey anti-rabbit antibody was added, and incubated for an additional 1 h at 37 °C. Finally, slides were washed, dried, coverslipped and viewed under a fluorescence microscope (×400).

### Cell culture

EPCs (CD34+ cells from cord blood) were isolated from cord blood from granulocyte-colony stimulating factor (G-CSF)-mobilized leukopheresis samples on the basis of CD133 expression, using an antibody-coupled magnetic bead cell isolation system (Stemcell Technologies, Vancouver, BC, Canada) as previously described [16]. Human umbilical cord blood was collected by the method of Moore et al. [17], as previously described [16]. To confirm purity of the EPCs, isolated cell populations were subjected to flow cytometry analysis as described previously [18, 19]. EPCs with appropriate cell markers (CD34+, CD133+, CD14-) were used in cell signaling studies.

Human dermal microvascular ECs (HMVECs) as well as all fibroblasts were collected from human tissues, which were digested in a cell culture media supplemented with FBS, collagenase, and hyaluronidase as done previously [20–22]. HMVECs were isolated from skin biopsies while the fibroblasts were isolated from synovial tissues of arthritis patients obtained at arthroplasty or synovectomy. HMVECs were isolated and purified using CD31 microbeads (Miltenyi Biotec, Bergisch Gladbach, Germany). HMVECs were grown in EBM-2 (Lonza, Walkersville, MD, USA) and were placed in reduced-serum EBM-1 before stimulation. EBM-1 without serum was used for HMVEC stimulations. EPCs were grown and stimulated in StemSpan SFEM (Stemcell Technologies) with no added supplement. Fibroblasts were grown in RPMI-1640 (Thermo Fisher Scientific) supplemented with 10 % FBS and were stimulated in serum free RPMI-1640.

### Id1 enzyme-linked immunosorbent assay (ELISA)

HMVECs, EPCs, monocytes, and RA, OA, and NL synovial fibroblasts were plated in 6-well plates at 200,000 cells/well and serum starved overnight. Media was exchanged and collected after 24 h. The collected media was analyzed by ELISA for Id1 (MyBioSource, San Diego, CA, USA). Manufacturer protocols were followed. SFs of RA, OA, and several other diseases were also analyzed by this ELISA. Both synovial and dermal fibroblasts were stimulated with varying cytokines and concentrations. Cytokines used were tumor necrosis factor alpha (TNF-α, Thermo Fisher Scientific), chemokine (C-X-C motif) ligand 16 (CXCL16, R&D Systems, Minneapolis, MN, USA), interleukin 17 (IL-17, R&D Systems), and TGF-β (R&D Systems). These cytokines were chosen because they are known to be upregulated in RA ST [23, 24]. The supernatant and exosome fractions were collected after 24 h and analyzed by this ELISA.

### RNA extraction and quantitative real-time polymerase chain reaction (RT-PCR)

Total RNA was isolated from synovial fibroblasts and HMVECs using RNAeasy mini RNA isolation kits in conjunction with QIAshredders (Qiagen, Valencia, CA, USA) following the manufacturer’s protocol. Following isolation, RNA was quantified and checked for purity using a Nanodrop spectrophotometer (Thermo Fisher Scientific). cDNA was then prepared using a Verso cDNA kit (Thermo Fisher Scientific) as per the manufacturer’s protocol. Quantitative PCR (qPCR) was performed using Platinum SYBR Green qPCR SuperMix-UDG (Thermo Fisher Scientific) following the manufacturer’s protocol. The primer pairs used were based on published sequences. Diluted cDNA was mixed with Platinum SYBR green qPCR SuperMix-UDG, forward and reverse primers specific for each gene (10 μM final concentrations), and incubated at the following cycles; 50 °C for 2 min, 95 °C for 2 min and 40 cycles of 95 °C for 30 s, 55 °C for 30 s and 68 °C for 30 s using an ABI Prism 7500 sequence detection system (Applied Biosystems, Foster City, CA, USA). The primers for human Id1 [25], are forward: AGAACCGCAAGGTGAGCAA and reverse: CCAACTGAAGGTCCCTGATGTAG. The primers used for β-actin were used previously, [12, 26] and are forward: GCTAGGCAGCTCGTAGCTCT and reverse: GCCATGTACGTTGCTATCCA. All samples were run in duplicate.

### Exosome purification

Exosomes were isolated from cell culture supernatants by differential centrifugation. RA synovial fibroblasts were plated at 200,000 cells/well in a 6-well tissue culture plate in serum-free media. Media was exchanged and incubated for 24 h after which the supernatant was collected. An aliquot of the supernatant was taken for later analysis and the remaining amount was subjected to several steps of differential centrifugation. All centrifugation was conducted at 4 °C. First, the supernatant was centrifuged at 300 × g for 10 min to remove free cells. The remaining supernatant was centrifuged at 10,000 × g to remove cellular debris, then at 30,000 × g to remove smaller debris. The supernatant was then transferred to an ultracentrifuge and spun at 110,000 × g overnight to pellet the exosomes. The supernatant was stored and the pellet was separated on an Optiprep density gradient (Sigma-Aldrich, St. Louis, MO, USA). The fractions containing exosomes were then isolated. Notably, exosomes isolated from RA and OA SFs were isolated similarly from rheumatoid factor-depleted SFs. The original whole supernatant, exosome and cellular debris-depleted fractions, exosome fractions, exosomes isolated from SFs as well as exosome fractions lysed with 0.5 % Triton X-100 (Sigma-Aldrich) were individually analyzed using the Id1 ELISA.

### Western blotting

HMVECs or EPCs were plated in 6-well plates at 500,000 cells/well and serum starved overnight. The wells were then stimulated with Id1 (Abnova, Taipei, Taiwan, 10 nM) over a 45-min time course. Cell lysates were prepared in reducing conditions and western blot analysis was done. Proteins were transferred from the gel to nitrocellulose membranes which were then blocked in 5 % nonfat dry milk in Tris-buffered saline + Tween 20 (TBST, pH 7.6). Membranes were probed with rabbit anti-human antibodies to both the phosphorylated (*p) and nonphosphorylated forms of Jnk, Erk_1/2_, PI3k, and P38 (Cell Signaling Technology, Danvers, MA, USA, 1:1000 dilution) in blocking buffer overnight at 4 °C. Membranes were washed three times with TBST and incubated with horseradish peroxidase (HRP)-conjugated donkey anti-rabbit antibody (Cell Signaling Technology, 1:1000 dilution) for 1 h at 25 °C. Membranes were washed with TBST and visualized using Pierce ECL Western Blotting Substrate (Thermo Fisher Scientific). X-ray film was used to visualize the blots after ECL. These films were scanned and bands were quantified by UN-SCAN-IT (Silk Scientific, Orem, UT, USA). Gel loading was accounted for by adjusting all phosphorylated data by the total amount of signaling protein, and fold change of the phosphorylated signal molecules was calculated with respect to the unstimulated lysate.

### Severe combined immunodeficiency (SCID) Matrigel plug angiogenesis assay

To examine the effects of the Jnk pathway in angiogenesis with Id1 stimulation in vivo, SCID mice were injected subcutaneously with sterile Matrigel (Corning Life Sciences, Tewksbury, MA, USA, 500 μL/injection) containing Id1 (10 nM) and Jnk-silenced HMVECs. HMVECs were silenced using silencing RNA (siRNA) inhibiting the Jnk signaling molecule (Santa Cruz Biotechnology, Santa Cruz, CA, USA). Either Jnk siRNA or nonspecific siRNA was transfected into the HMVECs using the TransIT-TKO Transfection Reagent (Mirus Bio, Madison, WI, USA). Manufacturer’s protocols were followed for the transfection. Twenty-four hours after transfection, some of the cells were lysed to confirm Jnk knockdown and the rest were injected into mice together with the Matrigel (2.5 × 10^5^ cells/injection). The Matrigel plugs were removed 5 days later and weighed. Hemoglobin (Hb) analysis was conducted on the plug homogenates as a measure of angiogenesis. Hb levels were measured by adding 25 μL of homogenate mixed with 25 μL of 3, 3′, 5, 5′-tetramethylbenzidine (TMB) reagent to 96-well plates. Finally, samples were incubated at room temperature for 5 min. Absorbance was read with a microplate reader at 450 nm. Hb concentration was determined by comparison with a standard curve in mg/mL. Hb concentration, after normalization with plug weight, is a reflection of the number of blood vessels in the tissue [27].

### Statistics

#### Statistical analysis

Results are expressed as the mean ± standard error of the mean (SEM). Data were analyzed using a Student’s *t* test. *P* values less than 0.05 were considered significant. For histology data on cryosections from human and rodent synovial tissues, all cells were considered for positive or negative Id1 staining by a board-certified pathologist blinded to the experimental setup. The results are presented as the percentage of positive cells in each section. At least three sections were evaluated (for both mouse and human specimens) and the results were averaged, then pooled in the respective groups for all tissues evaluated.

## Results

### RA ST fibroblasts express Id1

**Fig. 1:**
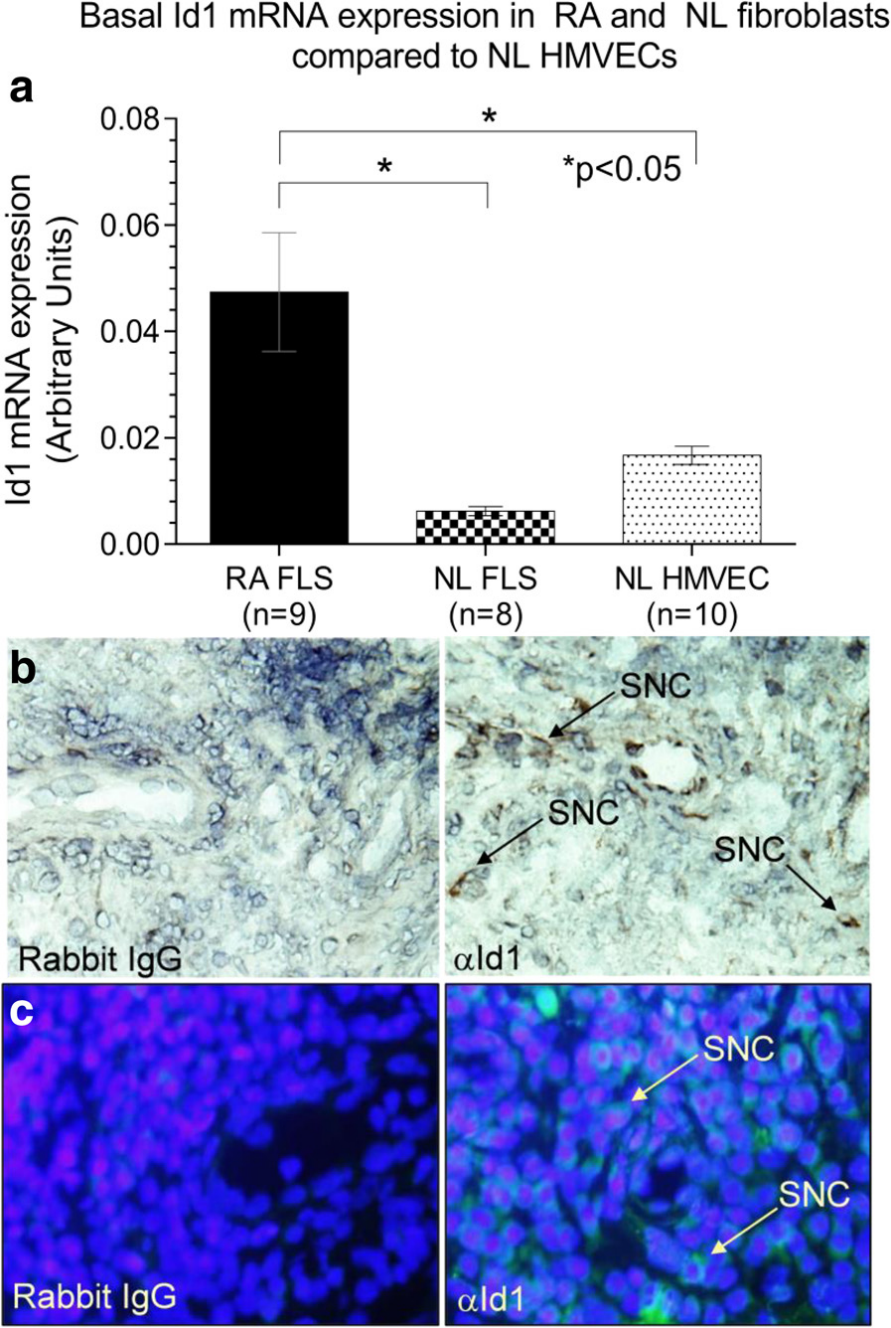
Id1 is expressed in ECs and ST fibroblasts. **a** mRNA was isolated from HMVECs and fibroblasts were isolated from NL and RA ST. mRNA was reverse transcribed into cDNA and underwent PCR for 40 cycles. RA fibroblasts showed significantly elevated Id1 expression compared to NL ST fibroblasts and HMVECs. **b** Id1 is expressed in RA STs. IHC was performed on RA, OA, and NL ST cryosections. Tissues were blocked and then incubated using a mouse anti-human Id1 (Abcam) primary antibody. After washing, tissues were incubated with a biotinylated anti-mouse secondary antibody (Vector Laboratories). Tissues were washed and subsequently developed with the Vectastain ABC kit (Vector Laboratories). Id1 is found on synovial cells (SNC) in the RA ST. **c** For immunofluorescence staining, RA, osteoarthritis (OA), and normal (NL) ST sections were fixed in cold acetone for 30 min. The STs were blocked with 5 % donkey serum and 20 % fetal bovine serum (FBS) in PBS at 37 °C for 1 h, and then incubated with rabbit anti-human Id1 antibody (Abcam, 10 μg/ml) or purified nonspecific rabbit IgG for 1 h at 37 °C in blocking buffer. The synovial tissues samples were washed with PBS, and a 1:200 dilution in blocking buffer of fluorescent-conjugated donkey anti-rabbit antibody was added and incubated for an additional 1 h at 37 °C. As shown, we could validate Id1 staining in RA ST similar to what was found using IHC (×400). *FLS* fibroblast-like synoviocytes, *HMVEC* human dermal microvascular endothelial cell, *Id1* inhibitor of DNA binding 1, *IgG* immunoglobulin, *NL* normal, *RA* rheumatoid arthritis, *SNC* synovial cell

Total RNA was isolated from nonstimulated ST fibroblasts and HMVECs. There was a significantly elevated Id1 mRNA expression in RA compared to NL ST fibroblasts and HMVECs, showing that Id1 production is upregulated in activated fibroblasts compared to NL ST fibroblasts or ECs (Fig. 1a).

### Immunohistochemical analysis of Id1 expression in mouse and human synovium

**Fig. 2:**
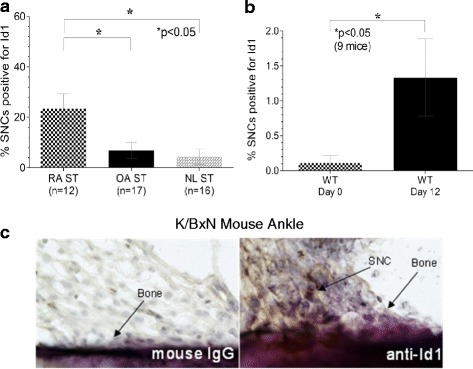
SNC Id1 expression is significantly higher in inflamed ST and in the ankles of K/BxN serum-induced mice. **a** Id1 expression was visualized on SNCs in ST by IHC. A significantly greater percentage of SNCs were positive for Id1 in RA compared to OA and NL ST. **b** Percentages of cells expressing Id1 were also evaluated on SNCs from joint tissues taken from K/BxN serum-induced mice. A significantly greater percentage of SNCs positive for Id1 compared to Wt (noninduced mice) was found. **c** Murine tissues were blocked and then incubated using a rabbit anti-mouse Id1 antibody (CalBioreagents). After washing, tissues were incubated with a biotinylated anti-rabbit secondary antibody (Vector Laboratories). Tissues were washed and subsequently developed with the Vectastain ABC kit (Vector Laboratories). Id1 is found on SNCs near the bone in the K/BxN mouse ankles. *Id1* inhibitor of DNA binding 1, *IgG* immunoglobulin, *NL* normal, *OA* osteoarthritis, *RA* rheumatoid arthritis, *SNC* synovial cell, *ST* synovial tissue, *Wt* wildtype

IHC staining for Id1 on RA, OA, and NL STs and K/BxN serum-induced mouse ankles indicated the presence of Id1 in these tissues. Id1 was highly expressed on SNCs of RA ST as well as in the K/BxN serum-induced mouse ankles (Figs. 1b, c and 2a–c). Immunofluorescence histology further validated the Id1 staining pattern seen with IHC (Fig. 1c). Analysis of these tissues by a pathologist revealed that RA STs had a significantly higher percentage of SNCs positive for Id1 than did OA and NL STs (Fig. 2a). Similarly, the day 12 K/BxN serum-induced mouse ankles had more Id1-positive SNCs than day 0 mouse ankles; although the difference was less striking (Fig. 2b and c). All images were taken at ×400.

### Effects of cytokines on fibroblast expression of Id1

**Fig. 3:**
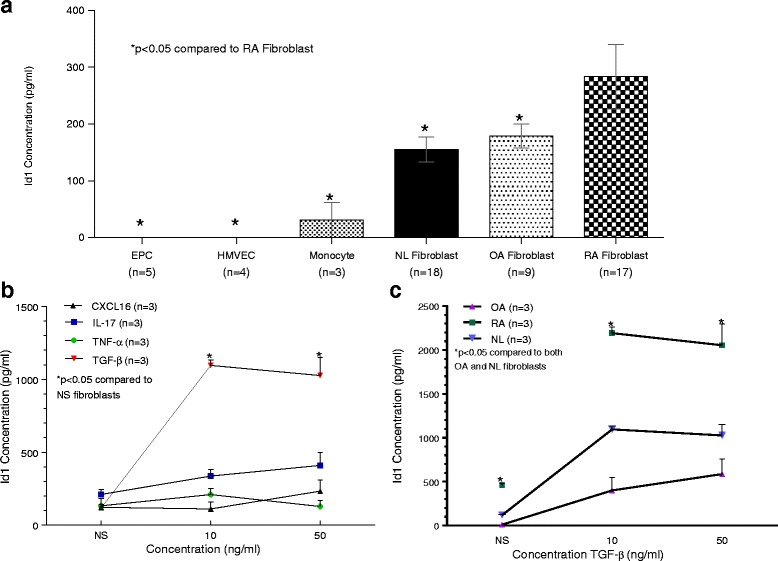
RA ST fibroblasts secrete Id1 and upregulate Id1 expression upon stimulation with TGFβ. **a** EPCs, HMVECs, monocytes, NL, RA, and OA fibroblasts were plated and serum starved. Cell culture media was replaced and the supernatants were collected 24 h later. The cell culture supernatants were examined for Id1 expression by ELISA (MyBioSource). EPCs, HMVECs as well as all fibroblasts were collected from human tissues, which were digested in a mix of cell culture media supplemented with FBS, collagenase, and hyaluronidase. HMVECs were isolated from skin biopsies while the fibroblasts were isolated from STs of patients with either RA, OA, or from NL patients. HMVECs were isolated and purified using CD31 microbeads (Miltenyi Biotec). All cell lines were between passages 3 and 6. No cytokine stimulation was used. **b** NL fibroblasts were plated and serum starved under the same conditions as panel a. Cell culture media with the respective cytokine was exchanged and collected 24 h later. CXCL16 (R&D Systems), IL-17 (R&D Systems), TNF-α (Life Technologies) and TGF-β (R&D Systems) were used at 10 ng/mL and 50 ng/mL concentrations. A not stimulated (NS) well was also used with no added cytokine (n = number of experimental replicates). **c** Synovial fibroblasts were plated under the same conditions as panel a. Cell culture media with TGF-β was exchanged and collected 24 h later. *CXCL16* chemokine (C-X-C motif) ligand 16, *EPC* endothelial progenitor cell, *HMVEC* human dermal microvascular endothelial cell, *Id1* inhibitor of DNA binding 1, *IL-17* interleukin 17, *NL* normal, *NS* nonstimulated, *OA* osteoarthritis, *RA* rheumatoid arthritis, *TGF-β* transforming growth factor beta, *TNF-α* tumor necrosis factor alpha

Analysis of supernatants of unstimulated cells of several types found in the RA synovium showed that synovial fibroblasts are the primary producer of Id1, and that RA fibroblasts produce more basal Id1 compared to NL and OA fibroblasts (Fig. 3a). Monocytes also produce some Id1, however much less compared to synovial fibroblasts. The ECs produced an undetectable amount of Id1. ELISA analysis of cell culture supernatants of NL synovial fibroblasts stimulated with various cytokines showed that TGF-β (at 10 and 50 ng/mL) and to a lesser extent IL-17 (at 50 ng/mL) increased Id1 production (Fig. 3b). TNF-α and CXCL16 had only minor effects on the secretion of Id1 by these cells. ELISA analysis of RA, OA, and NL synovial fibroblast cell culture supernatants stimulated with TGF-β at 10 and 50 ng/mL showed that TGF-β had a large effect on Id1 production by synovial fibroblasts (Fig. 3c). RA fibroblasts were significantly more sensitive to TGF-β stimulation with respect to Id1 production compared to OA and NL synovial fibroblasts.

### Identification of Id1 in fibroblast exosomes

**Fig. 4:**
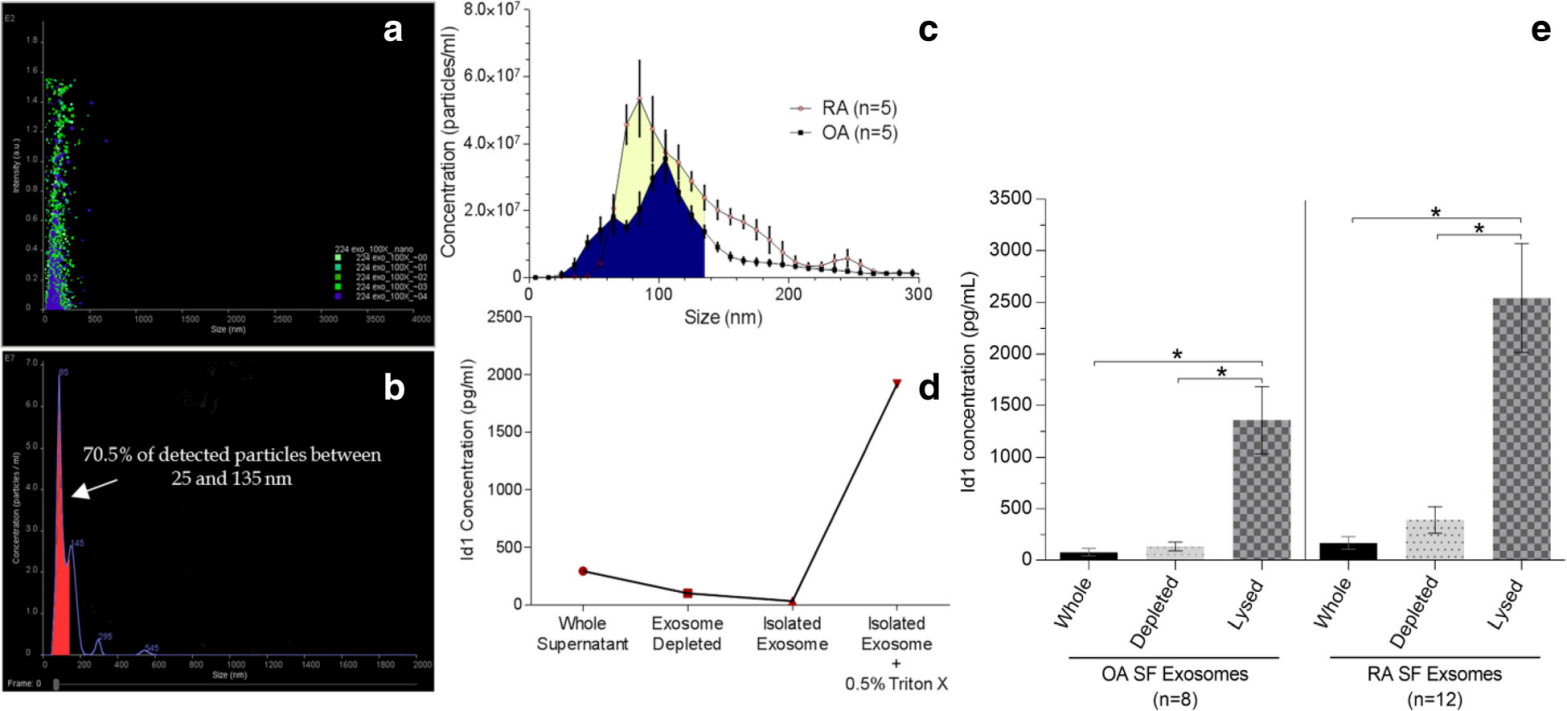
Id1 is contained in fibroblast exosomes. Fibroblasts from RA, osteoarthritis (OA), and normal (NL) STs were plated and maintained in CMRL medium supplemented with 20 % FBS, 2 mM L-glutamine, and 1 % penicillin/streptomycin in T175cm^2^ flasks and were used between passages 4 and 10. Cultures were moved to serum-free media DMEM/F-12 with Peprogrow serum replacement (Peprotech) for 2 days before harvesting. Fibroblast culture supernatants were concentrated to 1 mL (per flask) by centrifugation through an Amicon Ultracel 30 K filter (EMD Millipore, Billerica, MA, USA). Exosomes from cell supernatants were isolated and purified by serial ultracentrifugation. Nanoparticle Tracking Analysis was used to quantify the size and concentration of particles within the exosome fractions. **a** and **b** Cells were spun out at 1500 rpm for 5 min. Then the supernatants were cleared of heavier debris by spins at 10,000 × g for 30 min and 30,000 × g for 1 h. Exosomes were then spun down at 110,000 × g for 4–20 h. Exosome pellets were washed in PBS at 110,000 × g for 1.5 h – overnight. Some exosomes were further purified using a density gradient Optiprep (Sigma-Aldrich). Optiprep was diluted with PBS to produce the following layers: 5, 10, 15, 20, 30, 40, and 50 % w/v (densities of 1.031, 1.050, 1.084, 1.110, 1.163, 1.215, and 1.268 g/mL). The expected size of exosomes is between 25 and 135 nm and >70 % of the EVs isolated fall into this range (*red area*). **c** Representative diagram showing that we find more exosomes in RA (*yellow and blue areas*) compared to OA (*blue area only*) in fibroblast supernatants. **d** and **e** Whole and lysed (0.5 % Triton X-100) exosome fractions were then measured for Id1. We found that >80 % of the detected Id1 is contained within RA fibroblast cell supernatant exosomes. We similarly found that OA and RA SF have exosomes containing Id1 (n = number of sample wells measured for exosomes isolated from SF specimens from two separate patients for OA; and three separate patients for RA). All data was pooled in the respective OA and RA SF groups. *Id1* inhibitor of DNA binding 1, *OA* osteoarthritis, *RA* rheumatoid arthritis, *SF* synovial fluid

Analysis of fractions taken during a successive series of steps to isolate exosomes using differential centrifugation from RA synovial cell supernatants showed that the majority of the Id1 is found within exosomes when compared to the other fractions (Fig. 4). A small amount of Id1 was found in the whole supernatant and exosome-depleted fractions, and no Id1 was found in the unlysed exosome fraction. Triton X-100 at 0.5 % was used to lyse the exosomes and the majority of Id1 was found in this fraction. Notably, exosomes isolated from RA and OA SFs showed very low levels of Id1 unless the exosomes were disrupted by treatment with Triton X-100, confirming that Id1 is largely packaged within cellular exosomes and distributed outside of the cell (Fig. 2e).

### Western blot analysis of Id1-mediated signaling in ECs

**Fig. 5:**
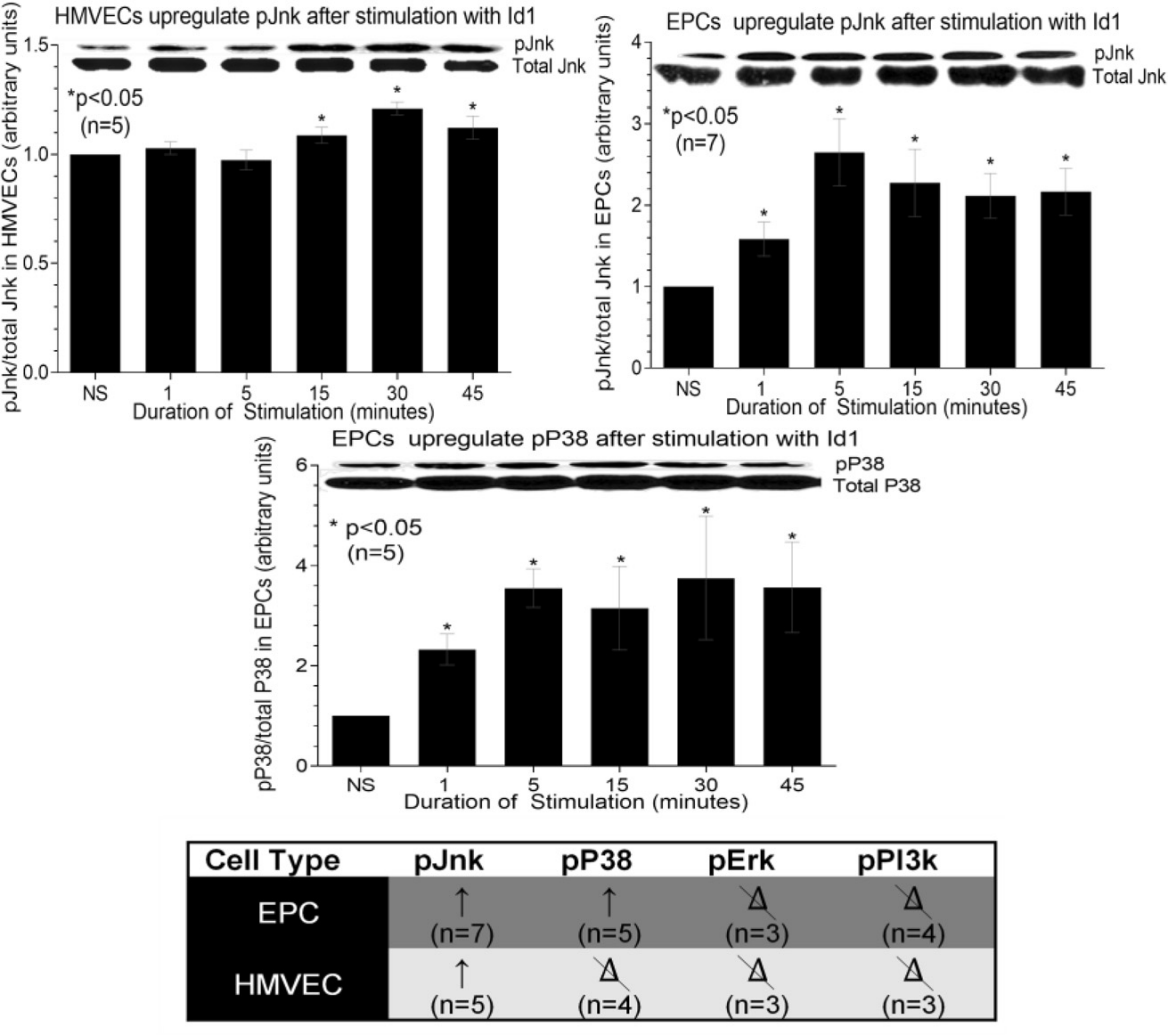
Id1 signals through the *pJnk pathway in HMVECs and EPCs. HMVECs and EPCs were cultured in 6-well plates and stimulated at different time intervals with human recombinant Id1. The cell lysate was collected and western blot analysis was performed. The results are shown as fold increase from the nonstimulated (NS), which was arbitrarily set at 1. The upper band represents phosphorylated signaling molecule (*p) and the lower band represents total signaling molecule. Using these bands, the amount of phosphorylated signaling molecule was quantified for each respective blot and results pooled. Upregulation (↑) of *pJnk and *pP38 was statistically significant in EPCs and upregulation of *pJnk was statistically significant in HMVECs. Upregulation of *pJnk plateaued at 5 min in EPCs and later in HMVECs at 30 min. Other signaling molecules were tested but results were not significant (data not shown, n = number of experimental replicates; the *delta symbol with slash* represents no change. *EPC* endothelial progenitor cell, *HMVEC* human dermal microvascular endothelial cell, *Id1* inhibitor of DNA binding 1, *NS* nonstimulated

Western blot analysis of *pJnk in HMVECs and EPCs following stimulation with Id1 showed that *pJnk was upregulated after 1 min of stimulation with Id1, an effect that persisted for at least 45 min in EPCs. However, in HMVECs upregulation was delayed by 15 min and was less robust, but continued to 45 min (Fig. 5a and b). Analysis of *pP38 in EPCs showed that *pP38 was upregulated almost immediately after stimulation with Id1 and this effect persisted at 45 min of stimulation (Fig. 5c). *pErk and *pPI3k did not show any upregulation after Id1 stimulation in either EPCs or HMVECs and *pP38 did not show any upregulation in HMVECs after Id1 stimulation (Fig. 5e). Results are shown as fold increase from nonstimulated (NS) cells arbitrarily set at 1. Upper bands represent phosphorylated signaling molecules (p) and the bottom bands represent the total signaling molecule for each respective protein. Independent lysates were run for both phosphorylated and total amounts of signaling proteins, and a representative blot is shown above each graph in Fig. 5. Using these bands from each respective blot, the amount of phosphorylated signaling molecule was quantified and the results were pooled.

### Id1 induces angiogenesis in vivo

**Fig. 6:**
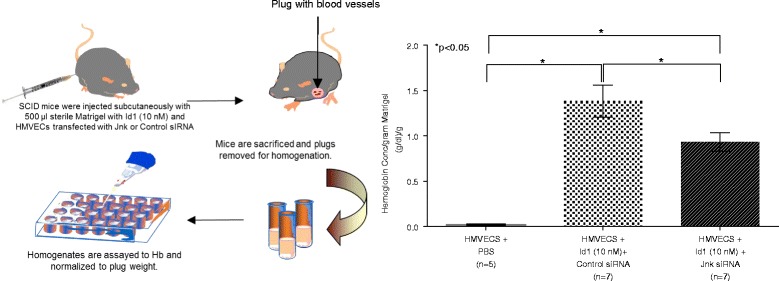
Jnk siRNA lowers HMVEC-mediated angiogenesis in the Matrigel plug assay. HMVECs were transfected with either control or Jnk siRNA designed to inhibit the Jnk signaling pathway. These cells were combined with 10 nM Id1 in Matrigel, which was injected subcutaneously into mice. Five days later, the Matrigel plug was removed, weighed, and homogenized. The hemoglobin assay was run to determine amount of hemoglobin in the plugs as a marker of angiogenesis. Jnk siRNA significantly inhibited angiogenesis in this assay. *HMVEC* human dermal microvascular endothelial cell, *Id1* inhibitor of DNA binding 1, *siRNA* silencing RNA

Hemoglobin analysis of Matrigel plugs injected into SCID mice with HMVECs and 10 nM Id1 showed less hemoglobin when the HMVECs were pretreated with Jnk siRNA compared to the control siRNA (Fig. 6). Hemoglobin concentration is representative of vascularization of the Matrigel plug. The results indicate that Id1 signaling through Jnk mediates the angiogenic effect of extracellular Id1.

## Discussion

Id1 is known to be a nuclear transcription factor characteristic of EPCs and cells that display a hyperproliferative phenotype. Our group has previously shown that Id1 is detected in RA synovium and SFs, and displays pro-angiogenic activity as an extracellular agonist [12]. Levels of Id1 are elevated in RA SFs and correlate with expression of the angiogenic chemokine CXCL16 (the ligand for the CXCR6 receptor) [12]. Correspondingly, CXCR6 knockout mice develop attenuated angiogenesis associated with profound decreases in arthritis progression and inflammatory cell recruitment to arthritic joints in K/BxN serum-induced mice. We now present evidence that synovial fibroblasts are largely responsible for the elevated amounts of Id1 in RA SF, and that approximately 80 % of the Id1 released by fibroblasts is packaged within exosomes. Moreover, pro-inflammatory cytokines known to be elevated in RA patients, such as IL-17 and especially TGF-β, induce significantly more fibroblast-derived Id1 in RA compared to OA or NL fibroblasts in vitro. Of special note, RA fibroblasts are significantly more sensitive to TGF-β stimulation than OA or NL fibroblasts with respect to Id1 production. These findings indicate that RA fibroblasts are primed to release Id1 in response to pro-fibrotic and angiogenic mediators, potentially due to epigenetic alteration of these cells in vivo.

Histologic analysis of ST revealed that Id1 is highly expressed on synovial SNCs and in the vasculature of the RA ST [12] in complete agreement with Sakurai et al., who showed Id1 and Id3 mRNA and protein are elevated in RA synovium [13]. Studies of knockout mice lacking expression of Id1 have shown its involvement in vasculogenesis and neuroblast differentiation in various cancer models normally characterized by extensive vascularization [28]. Tumors transplanted into these mice failed to grow and metastasize due to lack of proper tumor angiogenesis. Branching and sprouting of blood vessels in the neuroectoderm is also defective in double knockout mice lacking expression of Id1 and the related Id3, as these animals show premature differentiation of neuroblasts [28]. Thus it appears that Id1 expression is critical for angiogenic processes found in the tumor microenvironment or the RA joint, further suggesting that dysregulation of Id1 can lead to unwanted inflammatory outcomes. Furthermore, our findings provide additional evidence that Id1 may be used to assess or grade the severity of pathologic inflammatory conditions, as has been done previously for some cancers [29].

One of the many interesting features of Id1 is its ability to repress inhibitors of angiogenesis. Previous studies showed that Id1 indirectly regulates angiogenesis through transcriptional repression of thrombospondin-1 (TSP-1) [30]. Interestingly, a recent study showed that TSP-1 is strongly expressed by RA ST fibroblasts induced by TGF-β [31], both at the transcriptional and translational levels. This finding could raise questions about the role for Id1 repression of TSP-1. One possible explanation for this discrepancy may be that TGF-β could directly induce TSP-1 expression in RA, potentially overriding any transcriptional control exerted by Id1. Moreover, TSP-1 has also been shown to upregulate TGF-β in experimental inflammatory glomerular disease. Thus it appears that TGF-β and TSP-1 may upregulate expression of the other, making it tempting to speculate that upregulated Id1 serves as a negative control mechanism for TSP-1 and TGF-β expression [32].

The expression of diverse genes involved in remodeling of the extracellular matrix, angiogenesis, and intracellular signaling are also affected by Id1 [30]. Furthermore, Id1 represses p21 expression [4] to control EPC growth and maturation in the BM. Because of the ability of Id1 to downregulate expression of these potent repressors, it was reported that Id1 can function as an effective pro-angiogenic mediator produced by EPCs and pluripotent stem cells [33]. This idea was reinforced by reports identifying Id1 and Id3 as negative regulators of pluripotent stem cell maturation [11]. Because of its potent regulatory control of angiogenic and vasculogenic processes, it was not surprising to find that Id1 acts as a negative transcriptional regulator for proteins associated with malignant melanoma development [34, 35].

It is well documented that Id1 is a regulatory nuclear protein, however, we and others have previously shown that Id1 can be detected on the ECs in the RA synovium and in soluble form in synovial effusions [12, 13]. This suggests that Id1 travels outside the nucleus, but the functionality and method of this transfer has not been previously described. One possibility is that Id1 may be packaged into EVs such as cellular exosomes that can be released into the joint space or directly into adjacent cells. To test this possibility, we isolated EVs from RA synovial fibroblast supernatants known to contain Id1, and measured Id1 in the EVs and soluble protein fractions. We used differential ultracentrifugation to isolate EVs specifically around the size of exosomes. We visually confirmed a band at the density gradient between fractions 4 and 5, where exosomes would be expected (density between 1.084 g/mL and 1.163 g/mL). To more clearly define where Id1 is located, we further subdivided the EV fraction over a discontinuous Optiprep density gradient with seven fractions from 1.268 g/mL to 1.031 g/mL and collected the fractions containing exosomes. Id1 was barely detected on the surface of exosomes or on other EVs of similar density. However, addition of Triton X-100 (which lyses exosomes) revealed that >80 % of the detected Id1 is contained within exosomes, indicating that fibroblasts likely utilize exosomal mechanisms for Id1 export from the nucleus to the cytoplasm and out of the cell.

Id1 is also expressed at high levels in pro-B cells, but is downregulated in pre-B cells and mature B cells [36, 37]. Constitutive expression of Id1 in transgenic mice shows that these animals display severe defects in the development of B cells, demonstrating that cells in early development express Id1 and subsequently downregulate it as they acquire a mature phenotype [36]. It is possible that mature cells in the RA joint may take up fibroblast-derived Id1 (released within exosomes) to regulate cell proliferation in the inflammatory milieu of the RA synovium. Cellular crosstalk mediated by Id1 could thus transfer information from one cell to another despite inability of many inflammatory cells in the RA synovium to make Id1. Similarly, it was recently reported by Bourdonnay et al. that alveolar macrophages secrete the STAT-induced STAT signaling inhibitors SOCS1 and SOCS3 in exosomes and microparticles, respectively, for uptake by alveolar epithelial cells and subsequent inhibition of STAT activation in vitro and in vivo [38].

Kim et al. have generated transgenic mice in which Id1 is expressed specifically in T cells with the total number of thymocytes in these mice observed to be less than 4 % of that in Wt mice [39], again demonstrating the repressive nature of Id1 when overexpressed. Most cells were CD4-/CD8- double-negative cells bearing cell surface markers of multipotent progenitor cells, with apoptotic cells constituting about 50 % of the total thymocytes. Also of note, Tanaka et al. reported that the expression of Id1 in cardiac myocytes leads to the induction of apoptosis through a redox-dependent mechanism [40].

As previously noted, Id1 is upregulated in RA SF, and we show herein that Id1 initiates cell signaling events via an as yet unknown receptor. We did find that Id1 stimulates EPC signaling through *pP38 and *pJnk at almost all times measured, but not through Erk_½_ or PI3k. Peak upregulation of *pP38 and *pJnk was found at a stimulation time of approximately 5 min and persisted for at least 45 min. This is in contrast to HMVECs that displayed a more delayed and subtle response to Id1, showing significance in *pJnk expression after 15 min, then plateauing at 30 min. The kinetics of HMVEC and EPC signaling in response to Id1 with respect to *pJnk reveals differences in these cells, with the mature ECs showing a weaker response to Id1 than EPCs. Nonetheless, this experiment does show that it is possible for *pJnk to be a common signaling molecule in both EPCs and HMVECs when these cells are stimulated with Id1. This finding also identifies EPCs and ECs as cells able to bind Id1, further characterizing Id1 as both a vasculogenic and angiogenic mediator in the RA joint [12].

Locating where cell signaling events intersect in mature or progenitor ECs could be used to identify targets for Id1 inhibition and unwanted angiogeneic activity. We have performed similar signaling experiments on RA synovial fibroblasts as shown here for ECs and found that they signal through *pP38, *pJnk, *pJak2 and *pNF-κB in response to Id1 (data not shown), thus providing further evidence that Id1 may induce similar signaling events/pathways in various cell types. Of special note is the data (presented in Fig. 6) showing that inhibition of Jnk in HMVECs with siRNA significantly reduces the amount of Id1-induced Hb in the mouse Matrigel plug angiogenesis assay. Hb is a direct measure of angiogenesis, and we show that Jnk inhibition significantly reduced Id1-driven blood vessel formation in vivo. This finding demonstrates the feasibility of using signaling inhibition to disrupt essential pro-inflammatory functions (e.g., angiogenesis) initiated by Id1. Finally, it should be noted that the signaling and angiogenesis activity was shown using recombinant Id1, and not Id1 isolated from exosomes taken from RA SF or from fibroblast cell culture supernatants. In future studies, it would be interesting to examine the activity of exosome-derived Id1 for potential epigenetic alterations of the molecule, and if found, how they might affect Id1 activity.

The current standard of care for RA patients includes inflammatory cytokine inhibition. Hence, blockade of tumor necrosis factor-α (TNF-α) has resulted in improved pain control and decreased structural deformity in this disease. However, it is well documented that nearly one third of RA patients do not benefit from anti-TNF-α therapy. We posit that Id1 plays a central role in RA pathogenesis, independent of TNF-α, by expanding the vascular network. Overall, our data is suggestive that fibroblast-derived Id1 may contribute to vasculogenesis as well as angiogenesis by independent mechanisms, and that soluble Id1 can serve as either a biomarker or therapeutic target for angiogenesis in RA tissues.

## Conclusions

We show that Id1 is a pleiotropic nuclear protein exhibiting multiple functions including roles in angiogenesis, vasculogenesis, cell growth, and cellular self-renewal. We show that Id1 is released from fibroblasts and initiates cell activation, angiogenic and pro-inflammatory properties. We identify Id1 as a fibroblast-derived inflammatory protein capable of functioning as a signaling agonist, regulatory molecule, and angiogenic mediator in RA tissues.

## Abbreviations

AI: articular index
bHLH: basic helix-loop-helix
BM: bone marrow
CXCL16: chemokine (C-X-C motif) ligand 16
EC: endothelial cell
ELISA: enzyme-linked immunosorbent assay
EPC: endothelial progenitor cell
EV: extracellular vesicle
Hb: hemoglobin
HMVEC: human dermal microvascular endothelial cell
Id1: inhibitor of DNA binding 1
IgG: immunoglobulin G
IHC: immunohistochemistry
IL-17: interleukin 17
NL: normal
OA: osteoarthritis
RA: rheumatoid arthritis
RT-PCR: real-time polymerase chain reaction
SF: synovial fluid
siRNA: silencing RNA
SNC: synovial cell
ST: synovial tissue
TGF-β: transforming growth factor beta
TNF-α: tumor necrosis factor alpha
TSP-1: thrombospondin-1
Wt: wildtype
